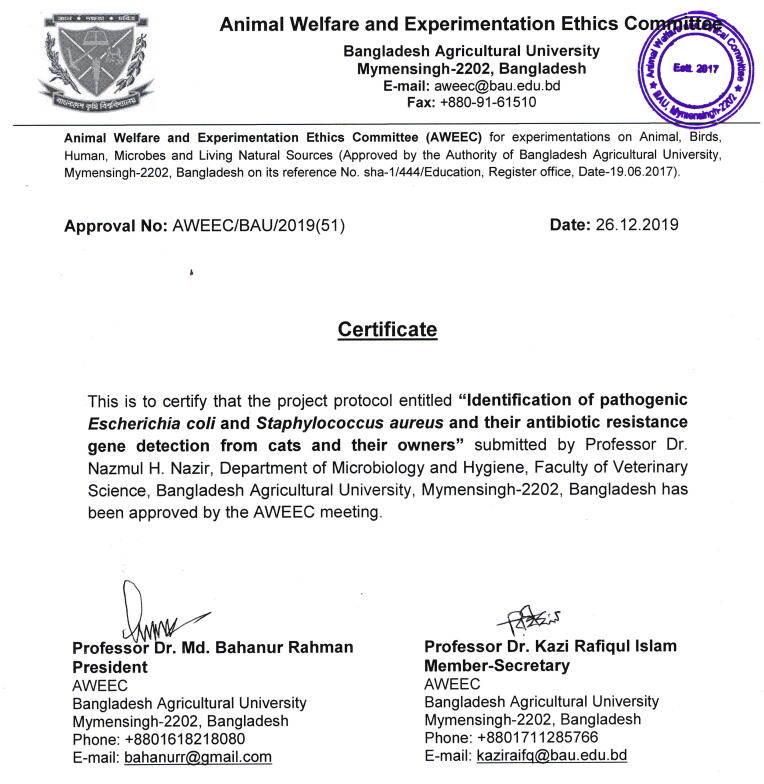# Domestic cats are potential reservoirs of multidrug-resistant human enteropathogenic *E. coli* strains in Bangladesh

**DOI:** 10.1016/j.sjbs.2023.103786

**Published:** 2023-08-23

**Authors:** Shanta Das, Ajran Kabir, Chandra Shaker Chouhan, Md. Ahosanul Haque Shahid, Tasmia Habib, Marzia Rahman, KHM Nazmul Hussain Nazir

**Affiliations:** a Department of Microbiology and Hygiene, Faculty of Veterinary Science, Bangladesh Agricultural University, Mymensingh 2202, Bangladesh; b Department of Medicine, Faculty of Veterinary Science, Bangladesh Agricultural University, Mymensingh 2202, Bangladesh

**Keywords:** Pet cat, Shiga-toxin, *rfbO157*, ESBL, MDR

## Abstract

Companion animals serve as our best friends, confidants, and family members. Thus, disease and antibiotic resistance gene transmission in pets and humans must be sought out. The study aimed to identify the common pathogenic *Escherichia coli* (*E.coli*) in pet cats and the antibiotic resistance patterns and resistant gene distribution. Samples (n = 210) were collected from different veterinary clinics in Bangladesh’s cities of Mymensingh and Dhaka. Pathogenic *E. coli* was identified using conventional and molecular approaches. The disc diffusion method assessed the resistance profile against 12 antibiotics, and PCR was used to identify the beta-lactam resistance genes. The prevalence of the *stx-1* gene was found to be 2.86%, whereas the *rfbO157* prevalence was found to be 1.90% in cats. The *stx-1* gene (n = 6) was 100% resistant to erythromycin and imipenem, whereas 100% sensitive to chloramphenicol. In turn, the *rfbO157* gene (n = 4) exhibited 100% resistance to erythromycin, imipenem, cefixime, and azithromycin. In addtion, we identified genes that exhibit resistance to beta-lactam antibiotics (100% *bla_TEM_*, 40% *bla_CTX-M_*, 40% *bla_SHV2_*). This study found shiga-toxin producing and extended-spectrum beta-lactamase (ESBL) producing *E. coli* for the first time in pet cats of Bangladesh. Furthermore, the antimicrobial resistance (AMR) profile of the isolated strains refers to the occurrence of multidrug, which concerns cats and their owners. The existence of these genes in non-diarrheic pet animal isolates indicates that domestic pets may serve as a reservoir for human infection. Thus, one health strategy comprising animal and human health sectors, governments, together with stakeholders is needed to confront multidrug-resistant *E. coli* infections in Bangladesh.

## 1 Introduction

*Escherichia coli* (*E. coli*) is one of the commonest members of gut microbiota that can affect both humans and animals. There are sixcategories of diarrheagenic *E. coli* as Enterotoxigenic (ETEC), Enteroinvasive (EIEC), Shiga toxin-producing (STEC), Enteroaggregative (EAEC), diffuse-adhering E. coli (DAEC) and Enteropathogenic (EPEC) *E. coli* (Puño-Sarmiento et al., 2013). EPEC *E. coli* causes infant diarrhea (Ochoa and Contreras, 2011, Hennessey et al., 2021). Strains of *E. coli* that produce STEC are the reason for an array of infections in people and animals, encompassing gastrointestinal and extra-intestinal difficulties like urinary tract infections. (Bentancor et al., 2007, Hu et al., 2021, Pan et al., 2021). Extra-intestinal pathogenic *E. coli*’s (ExPEC) involvement in causing severe infections has recently attracted attention regarding its zoonosis from a veterinary clinical standpoint (Bélanger et al., 2011, Pitout, 2012, Najafi et al., 2019). Hemolytic urinary syndrome (HUS), a worldwide distributed disease, is identified in a child closely aligning with a pet cat as a carrier of STEC O15:H7 strains (Rumi et al., 2012). Moreover, *E. coli* that produces Vero toxin (Shiga-like toxin) emerges to be predominant in the faces of animals, including cats that show no illness (Beutin et al., 1993). ETEC’s (Enterotoxigenic *E. coli*) presence in pets and their significance in diarrheal disease are poorly understood. Despite their widespread prevalence in dogs, a limited number of ETEC strains are currently studied in cats (Beutin, 1999). Enteroinvasive (EIEC) and Enteroaggregative (EAEC) strains are mainly tested for human infections, and the pathogenic significance of strains in diarrhea is unknown and requires further investigation. (Puño-Sarmiento et al., 2013).

The upsurge in antimicrobial resistance (AMR) is one of the imminent risks to medicine (Moo et al., 2020). Drug-resistant zoonotic illnesses can spread quickly to human populations through animals due to the growing usage of antibiotics in animals (WHO). Enterobacteriaceae, which produces the antibiotic-resistant enzyme ESBL, is a bit of a nightmare worldwide (Larramendy et al., 2021, Salinas et al., 2021., Ullah et al., 2023). ESBL enzymes hydrolyze third-generation of cephalosporins and aztreonam, although clavulanic acid inhibits them. This is now considered one of the most severe public health hazards. Moreover, the most frequent ESBL-causing strain is *E. coli* carrying *CTX-M* genes (Peirano and Pitout, 2019).

Cats were found to be present in 31% of U.K. residences, according to a survey conducted in 2007 (Murray et al., 2010). There are companion animals in over 58 million homes in the United States, with cats (59.1 million) being the most preferred (Enriquez et al., 2001). Similarly, petting animals, especially dogs, and cats, has become popular in Bangladesh, notably for youngsters’ and owners’ emotional and social well-being (Robertson et al., 2000). Due to this, more pets need treatments, and more significant amounts of antimicrobial are prescribed. *E. coli* is the principal repository of the resistance genes; therefore, inadequate constraints on antimicrobial have a significant role in the development of MDR strains posing a zoonotic risk to human health (Morato et al., 2009, Cui et al., 2022).

ESBL-producing *E. coli* (ESBL-EC) infections are becoming more prevalent in pets and humans (Ho et al., 2011, Cui et al., 2022). The feces of healthy cats and dogs are a significant reserver ESBL-EC (Cui et al., 2022). The overall development of antibiotic resistance rates among *E. coli* strains from farm and pet animals, as well as fish samples, has been the subject of numerous research studies all over the globe (Dos Santos et al., 2013, Schaufler et al., 2015, Chen et al., 2019, Kristianingtyas et al., 2020, Gruel et al., 2021., Ullah et al., 2023). In our country, cattle, sheep, and goats have been the subjects of the most exhaustive studies of *E. coli* infection (Johura et al., 2017). However, dogs and cats have recently resided near humans; consequently, the likelihood of pathogenic microorganism transmission to humans is extremely high. Therefore, some research must determine the abundance of AMR genes in *E. coli* isolates from domesticated pets. However, to our best knowledge, no published data are available in Bangladesh on identifying pathogenic *E. coli* strains and finding antibiotic resistance genes in cats and their owners. Considering the paucity, the research addressed pathogenic *E. coli* prevalence in domestic pet cats, their AMR pattern, and molecular detection of resistance genes.

## 2 Materials and methods

### 2.1 Ethic al approval

Current research proceeded in accordance with the guidelines of the Animal Welfare and Experimental Ethics Committee, Bangladesh Agricultural University. The samples (rectal swabs from cats) were obtained after getting the appropriate consent from cat owners and explaining the study’s objective. Approval No: AWEEC/BAU/2019(51).

### 2.2 Sample collection and processing

In total, 210 rectal swabs were obtained from pet cats enrolling in veterinary clinics in the communities of Dhaka and Mymensingh. The target population for this study is client-owned cats with access to a bed that visited veterinary clinics for routine exams, vaccinations, or other health complications with or without diarrhea. In order to circumvent self-contamination, we utilized sterile cotton buds and submerged the swabs in the sterile nutritional broth after collecting the swabs. Immediate transport to the lab facilitated overnight incubation at 37 °C of the obtained samples.

### 2.3 Isolation and identification

The enriched broth was diluted and inoculated onto Eosine Methylene Blue (EMB) (H.I. media, India) agar overnight at 37 °C. The colonies exhibiting classic *E. coli* cultural features were subcultured on selective media (EMB) to obtain purified isolates. Gram staining was used to confirm the morphology. Observing the cultural and Gram’s straining attributes, selected colonies were determined to perform several biochemical tests such as primary sugar (Sucrose, Maltose, Lactose, Mannitol, Dextrose) fermentation test, Coagulase test, Methyl-red test, Catalase test, Indole test, and Voges-Proskauers test (Farhad et al., 2021).

### 2.4 Molecular detection *E.coli* and virulence genes

Each *E. coli* strain’s genomic DNA was retrieved utilizing a boiling protocol (Hossain et al., 2013). Molecular detection was performed using genus specific primers listed in Table 1. Total 25 µl of PCR mixture was prepared where 12.5 µl of master mix (Promega, USA), 1 µl of forward and reverse primer (20 pmol/L) (Table 1), and 1 µl of DNA template was mixed in 9.5 µl of nuclease-free water were used. The results of PCR were filtered on a 1.2% agarose gel, tainted with Ethidium bromide, visualized with UV-transilluminator, and photographed. Confirmed E. coli isolates were then subjected to PCR for the confirmation of virulence genes including *rfbO157, stx-1,* and *stx-2 using* specific primers listed in Table-1.

**Table 1:** List of primers with sequences used in this study.

**Primer Name**	**Gene Targeted**	**Primer Sequences (5′-3′)**	**Amplic on size (bp)**	**Reference**
Eco-1	*malB*	5′GACCTCGGTTTAGTTCACAGA3′	585	(Nazir et al., 2005)
Eco-2		5′CACACGCTGACGCTGACC3′		
EC *stx-1*F	*stx-1*	5′CACAATCAGGCGTCGCCAGCGCACTTGCT3′	606	(Heuvelink et al., 1995)
EC *stx-1*R		5′TGTTGCAGGGATCAGTCGTACGGGGATGC3′		
EC *stx-2*F	*stx-2*	5′CCACATCGGTGTCTGTTATTAACCACACC3′	372	
EC *stx-2*R		5′GCAGAACTGCTCTGGATGCATCTCTGGTC3′		
*rfbO157*-F	*rfbO157*	5′AAGATTGCGCTGAAGCCTTTG 3′	497	(Farhad et al., 2021)
*rfbO157*-R		5′ CATTGGCATCGTGTGGACAG 3′		
*bla_TEM_*-F	*bla_TEM_*	5′CATTTCCGTGTCGCCCTTAT3′	793	(Ehlers et al., 2009)
*bla_TEM_*-R		5′TCCATAGTTGCCTGACTCCC3′		
*bla_ctx-M_*−F	*bla_CTX-M_*	5′ATGTGCAGYACCAGTAARGTKATGGC3′	593	(Ehlers et al., 2009)
*bla_ctx-M_* –R		5′TGGGTRAARTARGISACCAGAAYCAGCGG3′		
*bla_SHV2_*-F	*bla_SHV2_*	5′TTCGCCTGTGTATTATCTCCCTG3′	854	(Ehlers et al., 2009)
*bla_SHV2_*-R		5′TTAGCGTTGCCAGTGYTCG3′		

### 2.5 Antimicrobial susceptibility testing and detection of ESBL-producing *E. coli*

In order to ascertain the antibiotic susceptibility profile, twelve commonly used antibiotics for cats from seven antimicrobial classes were preferred. Antibiotic susceptibility testing was done among all genotypically confirmed *E. coli* isolates utilizing the standard agar disc diffusion method (Bauer et al., 1966). The antimicrobial assay used the commercially available Mueller Hinton Agar (H.I. media, India). Used antimicrobial agents were Erythromycin (E, 15 µg); Ampicillin (AMP, 10 µg); Cefuroxime (CXM, 30 µg); Cefotaxime (CTX, 30 µg); Cefixime (CFM, 5 µg); Norfloxacin (NOR, 10 µg); Chloramphenicol (C, 30 µg); Ciprofloxacin (CIP, 5 µg); Azithromycin (AZM, 15 µg); Gentamicin (CN, 10 µg); Imipenem (IMP, 10 µg); Florfenicol (FFC, 30 µg). Antimicrobial test findings are categorized according to the zone diameter interpretive criteria mentioned in the guideline of the Clinical Laboratory and Standards Institute (CLSI) (Clinical and Institute, 2017). MDR isolates have developed resistance to three or more different types of antimicrobial classes. Double-disk synergy assay was performed to determine the presence of ESBL-producing *E. coli* isolates according to previous study (Cui et al., 2022).

### 2.6 Molecular detection of antimicrobial resistant genes

The *E. coli* isolates underwent further testing for the presence of beta-lactamase producing genes including *bla_TEM_*, *bla_CTX-M_*, *bla_SHV2_*. The primers that were utilized in order to accomplish this PCR are shown in Table 1. After PCR, the product was illustrated as the procedure described before (Hossain et al., 2013).

## 3 Results

### 3.1 Occurrence of *E. coli* and virulence genes

**Fig. 1:**
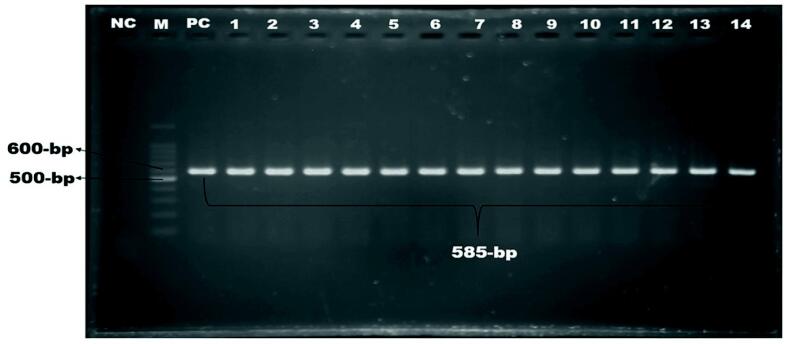
Amplification of malB gene (585 bp) of E. coli. Lane NC: Negative control, Lane PC: Positive control, Lane M: 100 bp DNA ladder (Thermofisher) Lane 1–6: Positive for malB gene of E. coli.

**Fig. 2:**
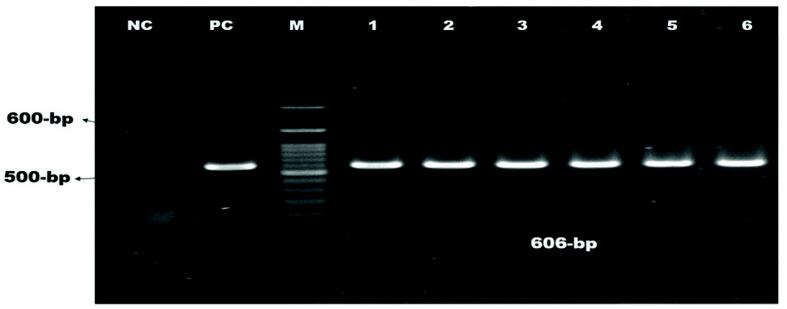
Amplification of *stx1* gene (606 bp) of E. coli. Lane NC: Negative control, Lane PC: Positive control, Lane M: 100 bp DNA ladder (Thermofisher) Lane 1–6: Positive for *stx1* gene of *E. coli*.

**Fig. 3:**
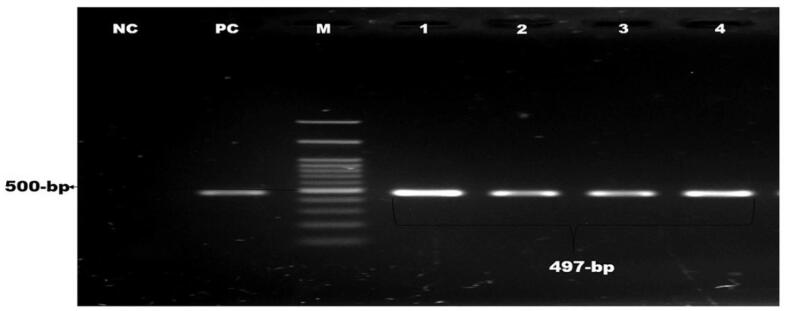
Amplification of *rfbO157* gene (497 bp) of *E. coli*. Lane NC: Negative control, Lane PC: Positive control, Lane M: 100 bp DNA ladder (Thermofisher) Lane 1–4: Positive for *rfbO157* gene of *E. coli*.

Of 210 isolates, 104 (49.52%) were confirmed as*E. coli* by PCR using the *malB* gene (Fig. 1). Six *malB*-positive *E. coli* isolates were containing *stx-1* gene and four were found containing *rfbO157* gene, but none contained *stx-2* or both genes. The overall occurrence of *stx-1* gene was 2.86% and *rfbO157* gene was 1.90% (Fig. 2, Fig. 3).). Intriguingly, the *stx-1* and *rfbO157* genes were found in 5.77 % and 3.85 % of *malB* positive *E. coli* samples, respectively. (Table 2).

**Table 2:** Occurrence of pathogenic *E. coli* (*stx-1 and rfbO157) gene* from domestic cats’ rectal swabs.

Pathogenic *E. coli*	No of total samples	No. of *malB* gene positive *E. coli* samples	No. of virulence gene positive *E. coli*	Occurrence of pathogenic *E. coli* in the total sample (%)	Occurrence of pathogenic *E. coli* in *malB gene* positive samples (%)
*stx1 gene*	210	104 (49.52%)	6	2.86	5.77
*rfbO157* gene			4	1.90	3.85

### 3.2 Antibiotic susceptibility profile and ESBL producing *E. coli*

**Fig. 4:**
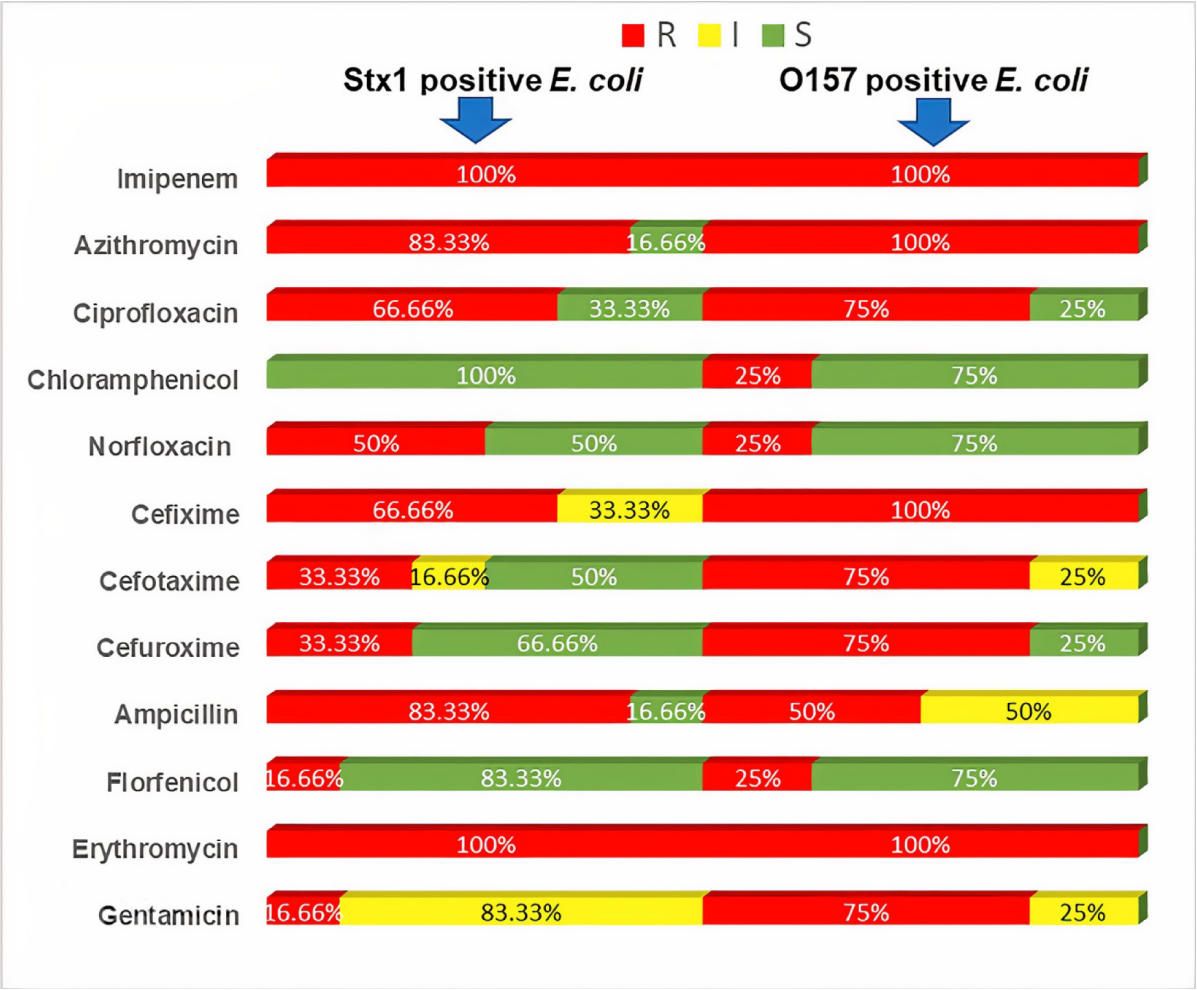
Antibiotic Susceptibility patterns of *stx1* and *O157* gene containing *E. coli* isolates.

Erythromycin and imipenem resistance was 100% in *stx-1* and *rfbO157*-positive *E. coli*. The pathogenic *E. coli* isolates containing *stx-1* gene were resistant to azithromycin and ampicillin (83.3%) but sensitive to chloramphenicol (100%), florfenicol (83.3%), and intermediate in gentamicin. Subsequently, the *rfbO157* gene containing *E. coli* isolates exhibited 100% resistance to cefixime and azithromycin, whereas it was 75 % sensitive to florfenicol and chloramphenicol (Fig. 4). Among ten virulence gene containing *E. coli* four were producing ESBL enzyme listed in Table-3.

### 3.3 Phenotypic MDR nature of pathogenic *E. coli*

All isolates of pathogenic *E. coli* exhibited the MDR phenotype. The most common MDR phenotype in pathogenic *stx-1* gene-containing *E. coli* was Erythromycin, Imipenem, Azithromycin, Ciprofloxacin, Ampicillin (E- IMP- AZM- CIP -AMP) (66.67%), followed by Erythromycin, Imipenem, Azithromycin, Ciprofloxacin, Ampicillin, Cefuroxime (E-IMP- AZM- CIP –AMP-CFM) (50%). In addition, one pathogenic *E. coli* isolates (R24) harboring the *stx-1* gene was resistant to all seven antimicrobial classes; another (R19) was resistant to nine antibiotics. (6 antimicrobial classes) (Table 3**)**. However, pathogenic *E. coli* with the *rfbO157* gene exhibited the most frequent phenotypic resistance pattern to erythromycin, imipenem, azithromycin, and ciprofloxacin (E- IMP- AZM- CIP) (100%). One pathogenic *E. coli* isolates (R 42) with the *rfbO157* gene resisted nine antibiotics (6 antimicrobial classes).

**Table 3:** Distribution of antibiotics resistant phenotypes and resistance genes among isolated *E. coli.*

***Stx-1* gene containing *E. coli***			
**Isolate**	**Resistant Phenotypes**	**ESBL Production**	**Resistant genotype**
R19	E, IMP, AZM, CIP AMP, CFM, CTX, CXM, CN	Yes	*bla_TEM_, bla_CTX-M,_ bla_SHV2_*
R24	E, IMP, AZM, CIP, AMP, CFM, NOR	Yes	*bla_TEM_, bla_CTX-M,_ bla_SHV2_*
R25	E, IMP, AMP, CXM	No	*bla_TEM_*
R26	E, IMP, AZM, CIP, AMP, CFM, CTX, NOR	No	*bla_TEM_*
R29	E, IMP, AZM, CIP, AMP, NOR	No	*bla_TEM_*
R32	E, IMP, AZM, FFC, CFM	No	*bla_TEM_*
***rfbO157* gene containing *E. coli***			
R39	E, IMP, AZM, CIP, CFM, AMP, CTX, CXM	Yes	*bla_TEM_, bla_CTX-M_, bla_SHV2_*
R40	E, IMP, AZM, CIP, CFM, CN, NOR, C	No	*bla_TEM_*
R41	E, IMP, AZM, AMP, CFM, CTX, CXM, CN	No	*bla_TEM_*
R42	E, IMP, AZM, CIP, CFM, CTX, CXM CN, FFC	Yes	*bla_TEM_, bla_CTX-M_, bla_SHV2_*

* R = Rectal swabs, E = Erythromycin, IMP = Imipenem, AZM = Azithromycin, CIP = Ciprofloxacin, AMP = Ampicillin, CFM = Cefixime, CTX = Cefotaxime, CXM = Cefuroxime, CN = Gentamicin, NOR = Norfloxacin, FFC = Florfenicol C = Chloramphenicol.

### 3.4 Molecular detection of beta-lactamase genes in *E. coli*

**Fig. 5:**
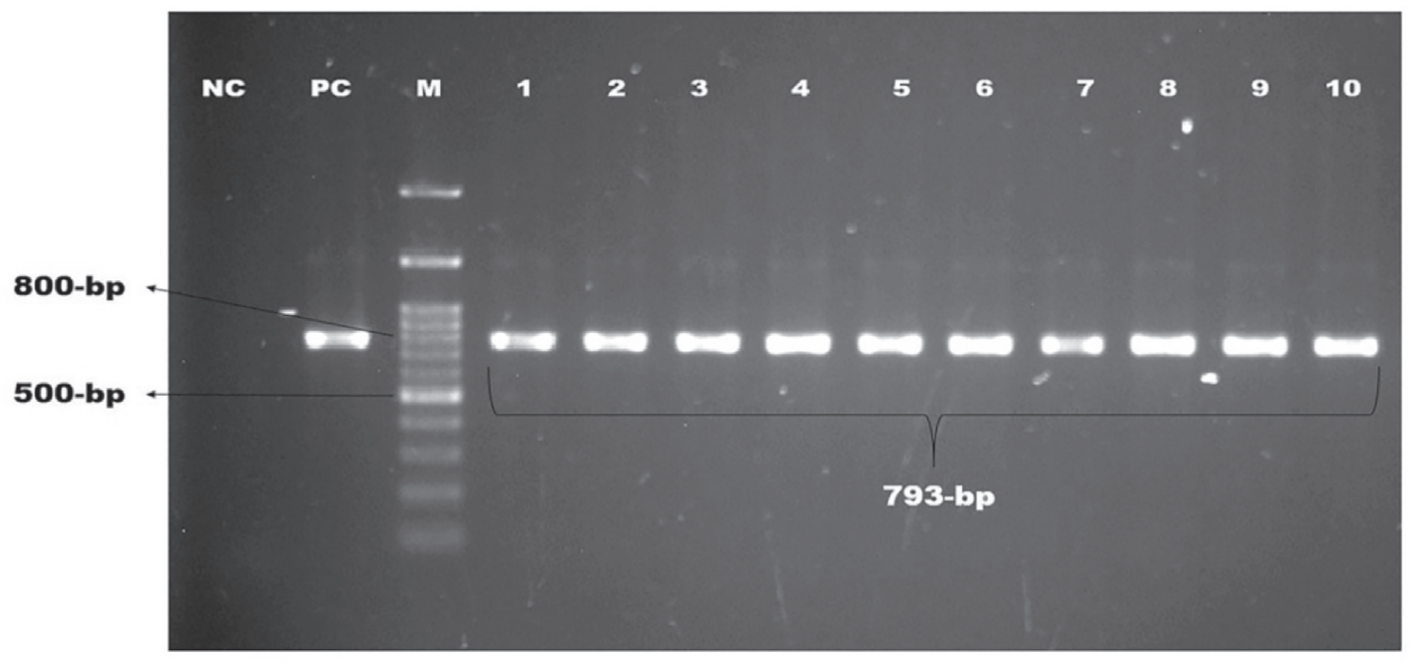
Amplification of *bla_TEM_* gene (793 bp) for beta-lactamase resistance. Lane NC: Negative control, Lane PC: Positive control, Lane M: 100 bp DNA ladder (Thermofisher) Lane 1–10: Positive for *bla_TEM_* gene of *E. coli*.

**Fig. 6:**
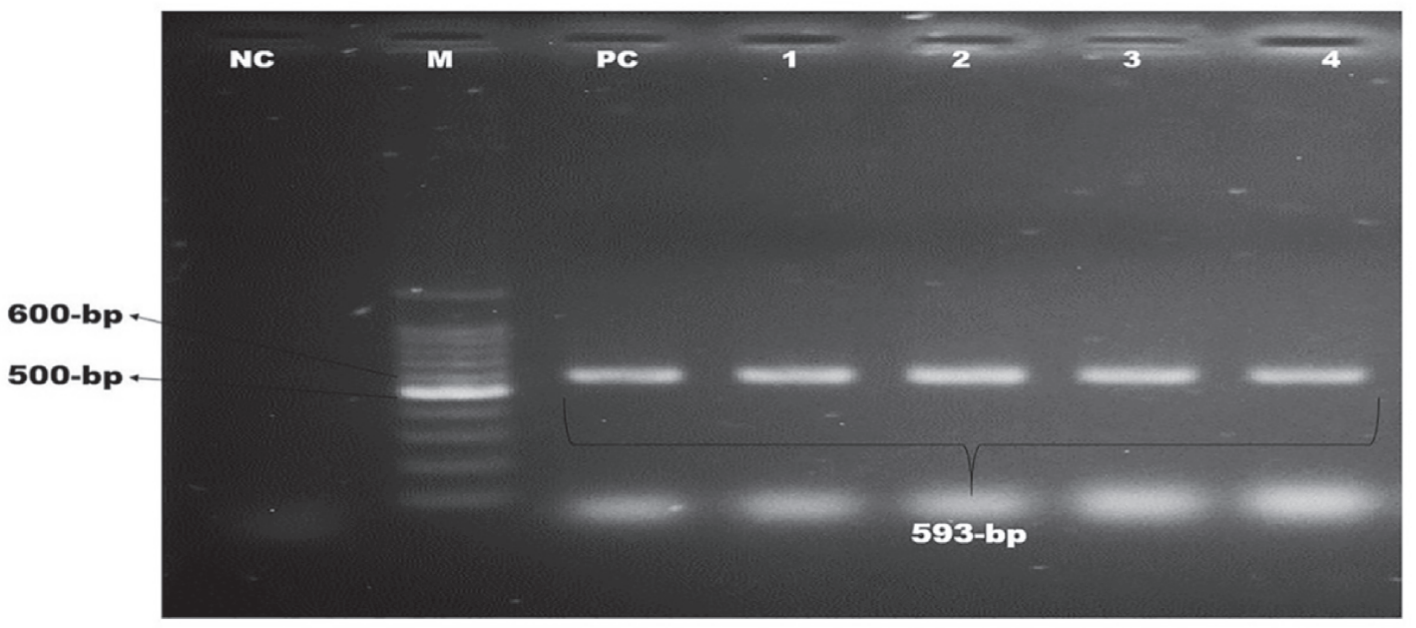
Amplification of *bla_CTX-M_* gene (593 bp) for beta-lactamase resistance. Lane NC: Negative control, Lane PC: Positive control, Lane M: 100 bp DNA ladder (Thermofisher) Lane 1–4: Positive for *bla_CTX-M_* gene of *E. coli*.

**Fig. 7:**
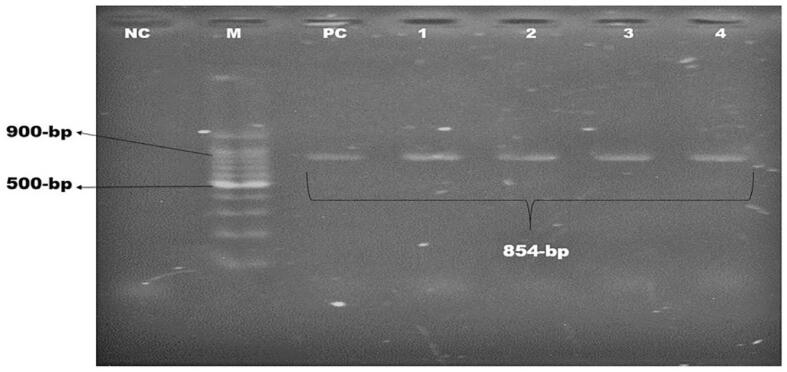
Amplification of *bla_SHV2_* gene (854 bp) for beta-lactamase resistance. Lane NC: Negative control, Lane PC: Positive control, Lane M: 100 bp DNA ladder (Thermofisher) Lane 1–4: Positive for *bla_SHV2_* gene of *E. coli*.

Each pathogenic *E. coli* containing *stx-1* and *rfbO157* genes had at least a single antibiotic resistance gene (*bla_TEM_*), and four of them possessed three resistance genes (*bla_TEM_, bla_CTX-M_, bla_SHV2_*) (Table 3). Molecular detection of these genes is depicted in Fig. 5, Fig. 6 & Fig. 7.

## 4 Discussion

The expansion of antimicrobial resistance in microbes from different animals has triggered a significant due to the risk of resistant infections and commensal bacteria being transmitted to humans. In recent years, there has been a rise in the number of people who keep pets as companions, which has increased the potential of zoonotic bacteria being passed from pets to humans (Cui et al., 2022). *E. coli* is an essential zoonotic agent linked to human and animal infectious diseases (Allocati et al., 2013, Bentancor et al., 2007). This study confirmed multidrug-resistant pathogenic *stx-1* and *rfbO157 E. coli* strains in pet cats, for the first time in Bangladesh.

This study revealed a 2.86% prevalence of STEC in pet cats. This *stx-1* gene incidence could be attributed to the habitat of cats living in an unhealthier environment and cats having outdoor assess roaming around in areas where pathogenic microorganisms are abundant and eating raw carcasses (Egberink et al., 2013). Cats can also be exposed to pathogenic microorganisms by consuming contaminated water (Srikullabutr et al., 2021). Moreover, wastes from households and the environment, like raw fish, meat, milk, vegetables, fruits, soil, water, etc., work as an essential medium for transmitting pathogenic enterobacteria to cats (WHO, 2020, Cairncross, 1990, Bach et al., 2002, Radi et al., 2014, Navab-Daneshmand et al., 2018).

On the other hand, the *rfbO157* genes were screened to detect the serotype and found a 1.90% prevalence among the cats.. The trend of offering companion animals raw meat-based diets (RMBDs) risks the transmission of these zoonotic pathogens (van Bree et al., 2018). Furthermore, these pathogenic strains of *E. coli* were predominantly detected in rectal swabs of cats, which could result from bacteria being shed via their feces (Ercumen et al., 2018, Navab-Daneshmand et al., 2018, Srikullabutr et al., 2021). Moreover, cats can serve as a repository of enteropathogenic *E. coli* even in the absence of diarrhea, which could better explain the availability of *E. coli* strains rectally (Morato et al., 2009).

Furthermore, *E. coli,* which produces Vero-toxin (Shiga-like toxin), was found in abundance in the feces of healthy domestic cats (Bélanger et al., 2011). Even so, animal-derived Extra-intestinal Pathogenic *E. coli* (ExPEC) could be considered zoonotic, meaning they can be transferred to people directly or indirectly (Bélanger et al., 2011). Similarly, a kid who lived near a pet cat was a carrier of STEC strains affected with the hemolytic urinary syndrome (HUS) that was previously reported (Rumi et al., 2012). As a result, the chance of contamination between humans and cats remained. However, none of this study’s STEC strains belonged to O157:H7 serotypes. They might belong to other enterohemorrhagic *E. coli* (EHEC) serogroups noticed in prior research (Abdulrazzaq et al., 2021).

In the antibiotic sensitivity test by disc diffusion method, isolates containing *rfbO157* and *stx1* genes were entirely resistant to erythromycin and imipenem. Additionally, complete resistance of Azithromycin and Cefixime was found in *rfbO157* isolates. Higher resistance of ciprofloxacin, ampicillin, cefuroxime, and cefotaxime was observed in the rest of the isolates, whereas Chloramphenicol and Florfenicol were found sensitive to all the isolates. The current study’s inferences coincide with those of prior studies (Madiha et al., 2015, Das et al., 2012, Puño-Sarmiento et al., 2013, Ali and Metwally, 2015, Liu et al., 2015, Saputra et al., 2017). This study shows that intermediate-resistance organisms can develop resistance at any time, which is concerning. On the other hand, chloramphenicol and florfenicol have proven to be the most efficient antibiotics against *E. coli* infection in cats (Carattoli et al., 2005, Johnson et al., 2009). This research observed MDR in all the pathogenic *E. coli* isolates. In total, three patterns of MDR were observed in which Erythromycin, Imipenem, Azithromycin, Ciprofloxacin, Ampicillin (E- IMP- AZM- CIP -AMP) (66.67%), and Erythromycin, Imipenem, Azithromycin, Ciprofloxacin, Ampicillin, Cefuroxime (E-IMP- AZM- CIP –AMP-CFM) (50%) were observed in stx1 gene containing *E.coli* and erythromycin, imipenem, azithromycin, and ciprofloxacin (E- IMP- AZM- CIP) (100%) observed in rfbO157 gene containing E. coli. This investigation discovered the MDR isolates of *E. coli*, which is concerning. Similar findings on the antimicrobial-resistant patterns of MDR-producing *E. coli* isolates have also been noticed earlier (Shaheen et al., 2010, Rzewuska et al., 2015, Chen et al., 2019, Piccolo et al., 2020).

This study used *bla_TEM_, bla_CTX-M_,* and *bla_SHV2_* genes to detect *E. coli* isolates that produce ESBL enzymes. Similarly to our findings, earlier research has documented the presence of multidrug-resistant ESBL-producing *E. coli* in cats and dogs (Zogg et al., 2018, Carvalho et al., 2021, Sfaciotte et al., 2021). Our pathogenic isolates were all *bla_TEM_* gene positive; four were *bla_TEM_, bla_CTX-M_*, and *bla_SHV_* genes positive. In prior research, these resistance genes were also found in cat feces (Toro et al., 2005, Weese et al., 2022). This is because cats have a close relationship with environmental elements, particularly water, and contaminated water is a significant factor in the spread of *E. coli,* which produces beta-lactamase among humans. Because of the negligent application of broad-spectrum antibiotics in animal feeds for treatment and prevention, these products may also pose a risk to pets (Wegener, 2003). Moreover, the sub-therapeutic usage of antibiotics in animals could also disseminate resistant organisms surrounding the environment, endangering animal and human health (Ejaz et al., 2021). The presence of ESBL-producing *E. coli* isolates in cats is alarming, and there remains a high chance of transferring these ESBL genes of *E. coli* to the owners and other people who come closer to them (Carvalho et al., 2016, Abbas et al., 2019).

## 5 Conclusions

The study accentuates the possible role of domestic cats in Bangladesh as reservoirs of ESBL-producing *E. coli* and multidrug-resistant Shiga-toxin-generating *E. coli* strains. The existence of multidrug-resistant *stx-1* and *rfbO157 E. coli* strains in pet cats is an enormous public health issue as these animals can act as carriers for antibiotic-resistant strains to spread into humans. In order to combat multidrug-resistant *E. coli* infections and Bangladesh’s rising antimicrobial resistance, governments, stakeholders, and the human and animal health sectors must work collaboratively.

## CRediT authorship contribution statement

**Shanta Das:** Conceptualization, Methodology, Writing – original draft. **Ajran Kabir:** Conceptualization, Methodology, Writing – original draft. **Chandra Shaker Chouhan:** Formal analysis, Writing – original draft. **Md. Ahosanul Haque Shahid:** Methodology. **Tasmia Habib:** Methodology. **Marzia Rahman:** Writing – review & editing, Supervision. **KHM Nazmul Hussain Nazir:** Writing – review & editing, Supervision.

## Declaration of Competing Interest

The authors declare that they have no known competing financial interests or personal relationships that could have appeared to influence the work reported in this paper.

## Figures and Tables

**Supplementary figure 1:**